# Ultrasound accuracy in evaluating renal calculi in Maysan province

**DOI:** 10.25122/jml-2023-0477

**Published:** 2024-02

**Authors:** Saud Kadhim Abbas, Thaer Saleh Sabor Al-Omary, Hayder Adnan Fawzi

**Affiliations:** 1 Department of Surgery, College of Medicine, University of Misan, Misan, Iraq; 2 Al-Mustafa University College, Baghdad, Iraq

**Keywords:** nephrolithiasis, reliability, stone location, hydronephrosis, stone size, CT, Computed Tomography, CI, Confidence Interval, ICC, Intraclass Correlation, IVU, Intravenous Urography, KUB, Kidney-Ureter-Bladder X-ray, US, Ultrasound

## Abstract

Renal calculi are a common clinical presentation. While ultrasound (US) is a widely used imaging modality for kidney stone diagnosis due to its accessibility and lower cost, its accuracy compared to computerized tomography (CT), the gold standard, remains understudied. This cross-sectional study evaluated the diagnostic accuracy of ultrasound for detecting and characterizing kidney stones compared to computed tomography (CT). Fifty-six patients with suspected kidney stones based on flank pain underwent abdominal ultrasound to assess stone presence, size, location, and the severity of any hydronephrosis (kidney swelling). These findings were then confirmed with a subsequent non-contrast CT scan. There was a fair agreement between US and CT (Kappa = 0.368) for detecting the stone location. The US could not detect 7 (12.5%) stones, being less sensitive in the middle and upper calyx compared to CT. There was a fair agreement between the US and CT (Kappa = 0.394) for detecting the severity of hydronephrosis. The US was less sensitive to moderate and severe hydronephrosis compared to CT. The abdominal ultrasound demonstrated excellent reliability for stone size measurement (intraclass correlation = 0.924), with CT measurements only slightly larger on average (mean difference 0.9 mm). Although abdominal ultrasound provides reliable stone size assessment, its capacity to accurately localize stones and assess hydronephrosis severity is limited.

## INTRODUCTION

Nephrolithiasis, often known as kidney stones, is the most prevalent disorder that can impact the renal system and urinary tract, impacting around 12% of the global population [1,2]. It results from crystals or crystalline concretion migrating throughout the genitourinary system from the kidney [1,2]. Typically, small calculi (2–3 mm) form inside the kidney cavity and should exit the human body through the urinary flow out of the urethral meatus with minimum pain. Calculi of greater size are troublesome and might require surgical intervention [3]. Around 80% of patients with nephrolithiasis have calcium stones, most of which are formed mostly of calcium oxalate or, to a lesser extent, calcium phosphate [4,5]. Cystine, uric acid, and struvite (magnesium ammonium phosphate) are the other major forms of kidney stones. Patients may have multiple crystal types of stones (e.g., calcium oxalate and uric acid) [6].

Several risk factors for kidney stones have been established, including family history and personal history of stone formation [7], decreased fluid intake [8], history of diabetes, obesity, gout, and hypertension [9], as well as several other urinary and dietary risk factors [3]. Patients with kidney stones might either be asymptomatic and incidentally diagnosed while performing an abdominal scan for several other symptoms, including abdominal, flank, or pelvic pain that is colicky and represents most of the acute presentation of kidney stones, dysuria, hematuria, fever, nausea, or vomiting [10].

Numerous factors, including genetics, geographical location, and socioeconomic status, significantly affect the incidence and composition of these stones in different world regions [11]. In the past, several researchers discovered a significant incidence of these stones in developing nations [11-16]. A meta-analysis by Liu *et al*. [17] found a 'stone belt' in West, Southeast, and South Asia, with a prevalence ranging from 5% to 19.1%. In contrast, most other regions, including East and North Asia, have a prevalence between 1% and 8%. Saudi Arabia had the greatest prevalence in Asia, ranging from 6.8% to 19.1% from 1989 to 2008 [17]. Similar trends of rising prevalence have been observed in western Iraq, although specific prevalence data is lacking [18]. Possible factors contributing to this condition include inadequate hydration, excessive or insufficient physical activity, obesity, bariatric surgery, or consumption of meals high in sodium or sugar. In certain individuals, infections and family history may hold significance. Excessive fructose consumption is associated with a higher likelihood of getting a kidney stone [19].

Low-dose computed tomography (CT) scans of the abdomen and pelvis are a highly accurate method for diagnosing kidney stones in people with a healthy body weight [20]. For individuals with a higher body mass index (BMI), standard-dose CT scans are typically preferred due to their ability to provide clearer images [21-23]. The sensitivity of CT in detecting kidney stones surpasses that of other existing modalities, with acceptable estimations indicating a rate of approximately 95% [24]. The limitations associated with CT encompass financial considerations and potential risks related to radiation exposure. Discussions related to expenses are sometimes complicated by the presence of several variables, such as charges, costs, and a refund, as well as the involvement of various stakeholders, including hospital systems, insurance firms, and the patient. According to Medicare data, CT scans for kidney stones generally cost about twice as much as a renal ultrasound and around one-third the cost of a magnetic resonance imaging (MRI). While low-dose CT scans offer a less expensive option, their costs are often comparable to standard CT scans [23,25,26].

Ultrasound (US) is a valuable alternative for diagnosing kidney stones, particularly for pregnant women and children or when CT scans are not readily available. The US is considered a reliable point-of-care bedside diagnostic tool in detecting renal stones and hydronephrosis in some emergency departments without causing management delays [27]. The US may be a viable option to replace standard-dose native CT of the abdominal cavity and the pelvis. This reduces the cumulative radiation dosage for patients with renal stones experiencing numerous imaging sessions. US is less accurate and more variable than CT scan for detecting nephrolithiasis, which is the key disadvantage. Pooled data shows a US sensitivity of 0.70 (95% CI, 0.67–0.73) and a specificity of 0.75 (95% CI, 0.73–0.78) [28].

The main aim of this study was to determine the diagnostic accuracy of ultrasonography in detecting renal stones, measuring their size, and defining their location compared to a CT scan, which is considered the gold standard method in diagnosing renal stones.

## MATERIAL AND METHODS

### Study design, setting, and participants

**Figure 1:**
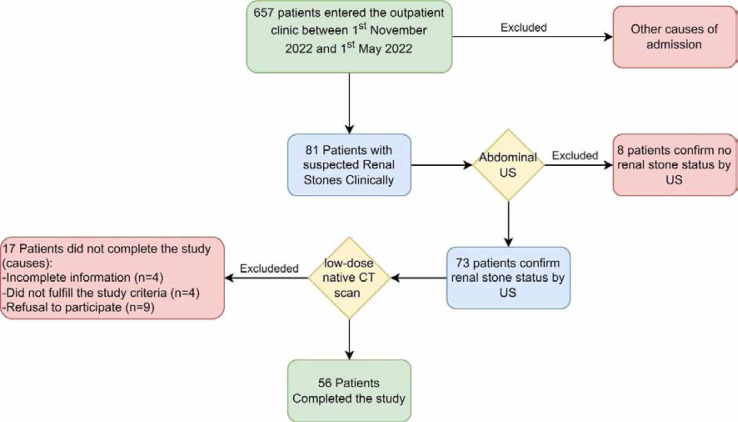
Flowchart of the study

This cross-sectional study was conducted at the radiology department of Al-Sadr Teaching Hospital in Misan, Iraq, between November 1, 2022, and May 1, 2022. The study involved 56 patients aged 19 – 81 years with clinically suspected renal stones. The patients were recruited from the outpatient clinic of the Al-Sadr Teaching Hospital and included adult patients aged ≥ 18 years of both genders with clinically suspected renal stones. Patients with solitary kidneys, chronic kidney disease, pregnant women, and patients who refused to participate in the study were excluded (Figure 1).

### Procedures

Demographic information, a comprehensive medical history, routine abdominal examination, and laboratory tests were conducted. An abdominal ultrasound was performed to assess for the presence of kidney stones. If stones were identified, their size, location, and any associated hydronephrosis (kidney swelling) were documented. Subsequently, the patients underwent a low-dose native CT scan of the abdomen and the pelvis. Similar to the ultrasound, the CT scan was used to confirm the presence of kidney stones, measure their size and location, and assess hydronephrosis or other abnormalities. The initial interpretation of both the ultrasound and CT scan images was performed by a single radiologist (first author) with five years of experience. The images were later sent to two independent radiologists to confirm the accuracy of the readings, who were blinded to the initial reports and confirmed initial reports (both had board certificates in radiology with five years of experience).

### Ultrasound techniques

The study used a gray-scale US (Voluson™ E6 GE HealthCare Technologies) with a 3–5 MHz curved transducer. All echogenic foci with acoustic shadow seen in the US renal pelvis or calyces were diagnosed as urinary tract stones. Secondary signs of obstruction, like hydronephrosis, hydroureter, nephromegaly, and perinephric stranding, were also noted, but only direct visualization of the stone was considered confirmatory. Stone detection by the radiologist was recorded, and if so, the investigator reviewed the US to obtain the maximum stone diameter.

### CT-scan method

The CT was obtained on a CT/multi-slice helical CT scanner (Siemens SOMATOM Sensation 64/ Siemens Healthineers). The exposure factors setting were KVp 130 and mAS 200–250. All scans were obtained from the upper border of the T12 vertebral body to the lower border of the symphysis pubis using 5 mm collimation without using oral or intravenous contrast material. Patients were placed in a supine position with the full urinary bladder at the time of the CT. The first author reviewed the CT scan results to obtain the maximum stone diameter and polar location within the kidney. CT scans were reviewed in the coronal and axial planes, and the largest diameter was utilized. In addition, hydronephrosis, renal masses, cysts, and anatomic abnormalities were recorded. The largest stone was then compared with findings from US imaging.

### Sample size

The sample size was determined using the G*Power version (3.1.9.7) [29,30]. We assumed a medium effect size of 0.5, an alpha level of 0.05, and a β-level 0.05 (power of detection), with a two-tailed (paired t-test). Based on these parameters, the calculated minimum sample size was 54 participants. We enrolled 56 patients in the study.

### Statistical analysis

All analyses used SPSS version 24.1 and GraphPad Prism version 10. The Shapiro–Wilk test was used to assess normality. A paired t-test was used to assess the differences between radiological modalities. Intraclass correlation (ICC) was used to assess the level of concordance, and the Bland–Altman plot was employed for further details. For categorical variables, the Kappa test was used. The significance level was considered significant if less than 0.05, and the *P* value was two-tailed.

## RESULTS

During the study period, 657 patients were admitted. However, only 56 fulfilled the inclusion criteria and underwent both US and CT scans. The study included 27 female participants (48.2%) and 29 male participants (51.8%), with a mean age of 46.8 ± 15.0 years. CT was considered the gold standard to confirm the diagnosis (Table 1).

**Table 1:** Demographic and kidney side

Variable	Value
*n*	56
Age (y), mean ± SD	46.8 ± 15.0
Sex, *n* (%)	
Female	27 (48.2%)
Male	29 (51.8%)
Kidney side, *n* (%)	
Left	30 (53.6%)
Right	26 (46.4%)

### Stone location

The agreement between the US and CT was 0.368 (Kappa value), which indicates fair agreement for detecting the stone location. US missed seven stones (12.5%) identified by CT. A more detailed analysis (Table 2) revealed a particular limitation of US: in the upper calyx, it detected only two out of seven stones found by CT. Conversely, in the middle calyx, the US falsely detected three stones that were not present in CT. Similarly, the US demonstrated reduced sensitivity in the lower calyx and pelvis regions, missing several stones in both locations. These findings suggest that US is less reliable than CT for detecting stones, particularly in the upper and middle calyx.

**Table 2:** Location of the stones according to radiological modalities

Parameters		CT scan				Total
		Upper Calyx	Middle Calyx	Lower Calyx	Pelvis	
US	None	3	0	4	0	7
	Upper Calyx	2	0	0	0	2
	Middle Calyx	2	2	4	5	13
	Lower Calyx	0	0	12	3	15
	Pelvis	0	1	4	14	19
Total		7	3	24	22	56

Kappa = 0.368, *P* value <0.001

### Hydronephrosis severity

The agreement between the US and CT was 0.394 (Kappa value), which indicates fair agreement for detecting the severity of hydronephrosis. The US could not detect hydronephrosis in 20 cases identified by CT. Furthermore, although the US accurately detected most cases of mild hydronephrosis (16 out of 17), it had limitations in diagnosing moderate and severe cases. US overdiagnosed moderate hydronephrosis (11 cases vs. 6 by CT) and underdiagnosed severe hydronephrosis (9 cases vs. 16 by CT) (Table 3). These findings suggest that US may not be as reliable as CT for assessing the severity of hydronephrosis.

**Table 3:** Detection of hydronephrosis severity according to radiological modalities

Parameters		CT scan				Total
		None	Mild	Moderate	Severe	
US	None	12	6	1	1	20
	Mild	4	8	3	1	16
	Moderate	1	3	2	5	11
	Severe	0	0	0	9	9
Total		17	17	6	16	56

Kappa = 0.395, *P* value <0.001

**Figure 2:**
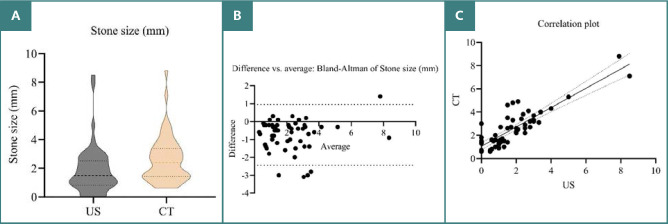
Comparative analysis of stone size measurements using US and CT imaging. A, Truncated violin plot; B, Bland – Altman analysis; C, Correlation plot of stone size by radiological modalities

There was excellent reliability between the US and CT for measuring stone size (ICC > 0.9). However, the stone size measurements in CT tended to be significantly higher than that measured by US (mean difference 0.9 mm), as illustrated by Table 4 and Figure 2.

**Table 4:** Assessment of agreement level in stone size between radiological modalities

US (mm)	CT (mm)	*P* value ^a^	ICC	*P* value ^b^
1.5 (0.8 – 2.5)	2.4 (1.53 – 3.38)	0.002	0.924	<0.001

a paired t-test; ^b^ intraclass correlation (ICC)

**Figure 3:**
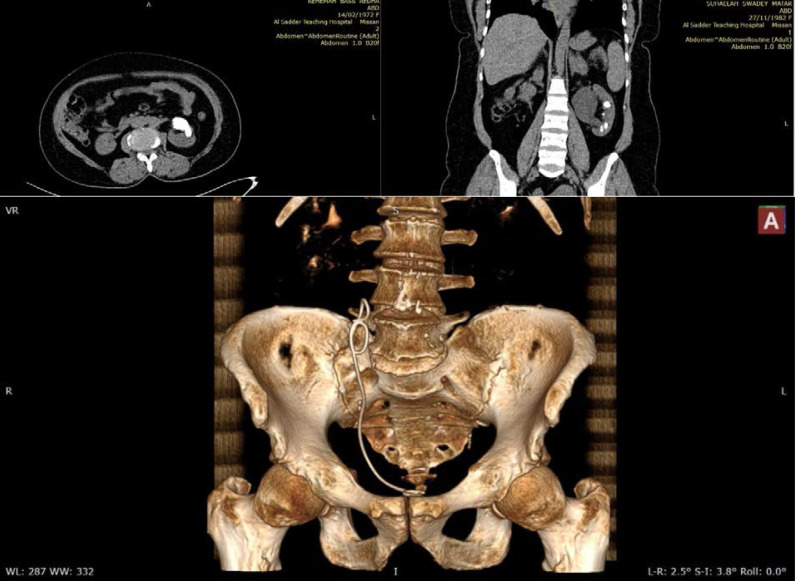
CT scan images of renal stone

**Figure 4:**
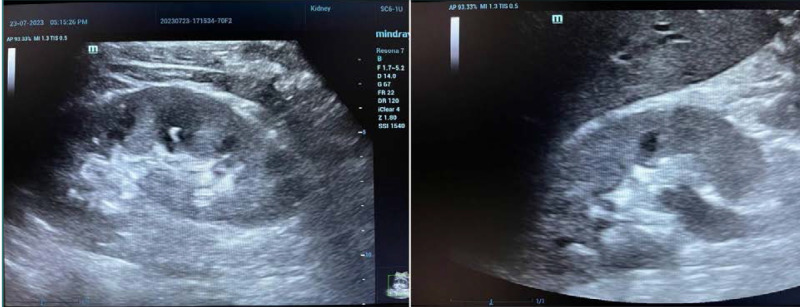
US image of renal stones

In the current study, US stone size measurement had excellent reliability; there was little tendency to lower the stone size compared to that measured by CT (since the mean stone size was lower in the US compared to CT) as shown in Figures 3 and 4. This lowering in measurement was more evident in stones with smaller sizes (as assessed by Bland–Altman analysis). A non-parametric assessment was used since the data did not follow a normal distribution. A truncated violin plot showed that the median stone size was significantly higher in CT compared to US, and most of the readings were concentrated in the lower range of stone size (the area of the widest width in the truncated violin plot). Bland–Altman analysis is a simple way to evaluate a bias between mean differences. The systematic bias was -0.7429 ± 0.8671 mm (95%CI, -2.442 to 0.9568), which indicates an underestimation of stone size by US compared to CT. This was more pronounced in small stones (1.5–4 mm). Scatter plots showed the direct significant correction between both stone sizes measured by US and CT scan, as illustrated in Figure 2.

## DISCUSSION

Ultrasonography is a frequently employed imaging method for evaluating the urinary tract. The utilization of this imaging modality offers numerous benefits compared to alternative methods, mostly due to its noninvasive nature and exceptional safety profile. These advantages stem from the absence of radiation exposure and the avoidance of intravenous contrast agents. Consequently, it is frequently advised to utilize renal US as the primary examination when assessing children and pregnant individuals suspected of urolithiasis. Additional benefits encompass affordability, superior image quality, and widespread accessibility [31]. Regrettably, this technique does possess inherent constraints regarding its applicability to renal calculi imaging. The existing body of literature has yielded inconsistent findings, particularly on the accuracy of the US in identifying renal calculi, with reported sensitivities ranging from 12% to 93% [32]. Additional research supported an enhanced sensitivity ranging from 77% to 79% when the US was employed with kidney-ureter-bladder X-ray (KUB) to assess ureteral colic [33,34]. However, the US is widely recognized for its limited proficiency in detecting stones in the mid-ureter [32]. Fowler *et al*. [35] found that the sensitivity of the US in detecting stones smaller than 3 mm was notably low, measuring only 13%. Moreover, this imaging technique tends to overestimate the dimensions of the stone due to the ambiguous delineation of the stone boundary in cases when renal or ureteral calculi are identified using the US. This overestimation can potentially impact the decision-making process on the appropriate management for the patient [36].

US demonstrated excellent reliability for stone size measurement compared to CT. However, US measurements tended to be slightly smaller than those by CT, with this underestimation being more pronounced for smaller stones (as assessed by Bland–Altman analysis). US may be less accurate than CT scans for determining stone location, particularly in the middle and upper calyx, and for detecting moderate to severe hydronephrosis, despite showing fair agreement between the two methods for overall stone detection. In contrast to CT scans, US examinations had a somewhat lower sensitivity and restricted specificity in detecting stones, with reported ranges of 24% to 70% and 88% to 94.4%, respectively [37-39]. Furthermore, it should be noted that the accuracy of stone sizing on US imaging is suboptimal, as it tends to overestimate the size of stones measuring 5 mm or smaller by an average of 3.3 mm [40]. The US is operator-dependent and lacks high-resolution devices, which reduces the stone size. Since stone size is crucial for determining both the likelihood of spontaneous passage and the most suitable surgical options, it is critical for clinical decision-making. Decisions made only based on stone size in the field of management lead to inaccurate guidance in around 22% of instances. Two of the biggest issues that hinder the broader adoption of US imaging are the need for improved stone detection and sizing accuracy [41].

Unenhanced helical CT is the preferred method for evaluating urinary calculi [42]. Due to its heightened sensitivity compared to conventional radiography and intravenous urography (IVU), CT scans have enhanced the identification of renal and ureteral calculi [43,44]. Upon conducting a comprehensive examination of the existing scholarly works, it has been determined that the diagnosis accuracy rates for acute ureteral colic are 100% for CT scans and 64% for IVU [44-47]. CT scans have predominantly supplanted IVU as the preferred method for assessing acute flank discomfort. Concerning the estimation of stone dimensions, Tisdale *et al*. [48] conducted a comparative analysis between transverse and craniocaudal measurements of 61 stones with a diameter of 1 cm. This analysis was performed using CT scans and KUB. The transverse dimensions showed a similarity between CT scans and KUB. However, CT scans overestimated the craniocaudal dimension by 1.4 mm compared to KUB [48].

The observational nature of the study (level of evidence 3b) and the relative sample size limits the generalizability of the findings. In addition, it was conducted at a single center and may introduce selection bias, limiting the generalizability to other institutions.The inclusion of women from diverse ethnic backgrounds is a strength, as it allows for some degree of generalizability to this specific population demographic.

## CONCLUSION

The US offers excellent reliability in detecting stone size. However, it has a fair ability to detect stone location and the severity of hydronephrosis. There is a small tendency to lower the stone size compared to that measured by CT. There have been debates about the accuracy of ultrasound in diagnosing nephrolithiasis, but it has significantly improved over the last several decades. It is now becoming the first imaging modality for evaluating patients with renal calculi. It is increasingly prominent in modern-day urology practice, helping clinicians determine the renal calculi size and site. The US is relatively sensitive and specific for diagnosing renal stones, but it lacks the sensitivity for the direct imaging of the ureteric calculi. Therefore, it is recommended to use a CT scan if ureterolithiasis is clinically suspected or US examinations are equivocal. The findings of this study can be validated by other studies on a larger cohort of patients.

## Data Availability

Zenodo: “Nephrolethiasis”. https://doi.org/10.5281/zenodo.10223421 Data are available under the terms of the Creative Commons Attribution 4.0 International Public License (CC0 1.0 Public domain dedication).
